# Inhibition of miR-10b treats metastatic breast cancer by targeting stem cell-like properties

**DOI:** 10.18632/oncotarget.28641

**Published:** 2024-08-26

**Authors:** Alan Halim, Nasreen Al-Qadi, Elizabeth Kenyon, Kayla N. Conner, Sujan Kumar Mondal, Zdravka Medarova, Anna Moore

**Affiliations:** ^1^Precision Health Program, Michigan State University, East Lansing, MI 48824, USA; ^2^Department of Radiology, College of Human Medicine, Michigan State University, East Lansing, MI 48824, USA; ^3^Department of Microbiology, Genetics, and Immunology, Michigan State University, East Lansing, MI 48824, USA; ^4^Veterinary Diagnostic Laboratory, College of Veterinary Medicine, Michigan State University, East Lansing, MI 48824, USA; ^5^Transcode Therapeutics Inc., Newton, MA 02458, USA

**Keywords:** breast cancer, metastasis, stem-like cells, nanoparticle, miR-10b

## Abstract

Despite advances in breast cancer screening and treatment, prognosis for metastatic disease remains dismal at 30% five-year survival. This is due, in large, to the failure of current therapeutics to target properties unique to metastatic cells. One of the drivers of metastasis is miR-10b, a small noncoding RNA implicated in cancer cell invasion, migration, viability, and proliferation. We have developed a nanodrug, termed MN-anti-miR10b, that delivers anti-miR-10b antisense oligomers to cancer cells. In mouse models of metastatic triple-negative breast cancer, MN-anti-miR10b has been shown to prevent onset of metastasis and eliminate existing metastases in combination with chemotherapy, even after treatment has been stopped. Recent studies have implicated miR-10b in conferring stem cell-like properties onto cancer cells, such as chemoresistance. In this study, we show transcriptional evidence that inhibition of miR-10b with MN-anti-miR10b activates developmental processes in cancer cells and that stem-like cancer cells have increased miR-10b expression. We then demonstrate that treatment of breast cancer cells with MN-anti-miR10b reduces their stemness, confirming that these properties make metastatic cells susceptible to the nanodrug actions. Collectively, these findings indicate that inhibition of miR-10b functions to impair breast cancer cell stemness, positioning MN-anti-miR10b as an effective treatment option for stem-like breast cancer subtypes.

## INTRODUCTION

Breast cancer is estimated to be the most diagnosed cancer overall and second-most lethal cancer among women in 2024 [1]. Changes to screening guidelines and advances in medicine have greatly increased survival rates; however, the most favorable prognoses are reserved for breast cancer detected when still localized, with a five-year survival rate of 99% [2]. Five-year survival rates for breast cancer that has metastasized to distant sites remain dismal at 30% overall [2]. A major contributor to this disparity in survival between localized and metastatic disease is the lack of therapeutics designed specifically for targeting metastases. Indeed, the breast cancer subtypes with the best survival rates – hormone receptor-positive or HER2-enriched – are those that are less likely to metastasize and with dependencies on signaling pathways that can be targeted therapeutically [2]. In contrast, metastatic disease is commonly of the triple-negative subtype, lacking a clear target and making treatment particularly difficult. While there is a need to identify treatments for triple-negative breast cancer in general, it is metastases that cause most patient deaths [3–5]. As such, the development of therapeutics aimed specifically at metastases or the metastatic process is of great clinical urgency. These therapeutics would serve as effective treatments for the most common cause of death not only for triple-negative breast cancer but for all aggressive breast cancers.

To fill this gap and develop effective options for treatment of metastatic breast cancer, it is important to understand the drivers of metastasis. MicroRNA-10b (miR-10b) is a small noncoding RNA molecule overexpressed in metastases compared to their primary tumors [6]. It has been implicated in breast cancer cell invasion and migration [7–9], and its overexpression is sufficient to confer onto breast cancer cells the ability to spontaneously metastasize [8]. Importantly, we found that miR-10b also serves as a critical driver of metastatic cell viability and proliferation [9]. This discovery led us to the notion that inhibition of miR-10b is a feasible mechanism to treat metastatic breast cancer. To accomplish this, we synthesized the nanodrug consisting of anti-miR-10b antisense oligomers (ASOs) conjugated to iron oxide-based magnetic nanoparticles (MN) that serve as delivery vehicles for oligonucleotides to tumor cells *in vivo.* The magnetic properties of these nanoparticles allowed for their detection by magnetic resonance imaging (MRI) so the delivery of the nanodrug can be monitored non-invasively [7]. Our previous studies in metastatic breast cancer models showed that systemic delivery of this therapeutic, termed MN-anti-miR10b, in mice bearing aggressive primary tumors prevented the onset of metastatic spread with high reproducibility, and if metastases were already present, their growth was halted [7]. Subsequent studies found that combination therapy with doxorubicin elicited regression and elimination of metastases in metastatic breast cancer models corresponding to Stage II and IV of human disease even after treatment was stopped [9, 10].

While MN-anti-miR10b shows tremendous clinical potential, a limitation of our previous studies is their focus on therapeutic outcomes. MiR-10b has over 350 predicted targets [11], many of which regulate gene transcription and translation themselves. Currently it is not known which genes and processes are governed by miR-10b inhibition by the nanodrug, but most importantly, it is not clear which properties make metastatic cells susceptible to the nanodrug actions. Thus, in this work we analyzed differentially expressed genes in response to miR-10b inhibition by the nanodrug to determine which biological processes are affected and how these processes are involved in the therapeutic response. Answering these questions and understanding the consequences and the mechanism of miR-10b inhibition in cancer cells may yield insights toward better therapy optimization for individual patient candidates and/or more effective adjuvant drug combinations.

Previously, we have observed various MN-anti-miR10b effects on cancer cells *in vitro*, including decreased migration, invasion, and proliferation [7, 9] and a direct effect on viability [9, 12]. Phenotypic effects were observed in as little as 24–48 hours treatment, with miR-10b expression decreased almost 90% relative to controls [7, 9]. In this study, we first determined the persistence of miR-10b inhibition after repeated treatments in mouse models of metastatic breast cancer. We observed an average of 99% downregulation within two weekly treatments, demonstrating the ability of MN-anti-miR10b to overcome physiological barriers and effectively downregulate its primary target. To identify mechanisms of therapy, we next performed RNA sequencing of breast cancer cells treated with MN-anti-miR10b *in vitro*. This revealed that inhibition of miR-10b affected many genes associated with developmental processes, suggesting that MN-anti-miR10b acts on the properties of cancer cells conferred by their dedifferentiation into a more stem cell-like state. We also found that this relationship was not limited to breast cancer and has a potential to be extended to other cancers. Lastly, we show that miR-10b expression is tightly connected to stem-like cancer cell subpopulations and demonstrate that inhibition of miR-10b decreases phenotypes associated with stemness. These data provide an explanation for the efficacy of MN-anti-miR10b in mouse models of metastatic breast cancer and support its use in high grade, poorly differentiated breast cancer cases.

## RESULTS

### Systemic administration of MN-anti-miR10b efficiently downregulates miR-10b in metastases

**Figure 1:**
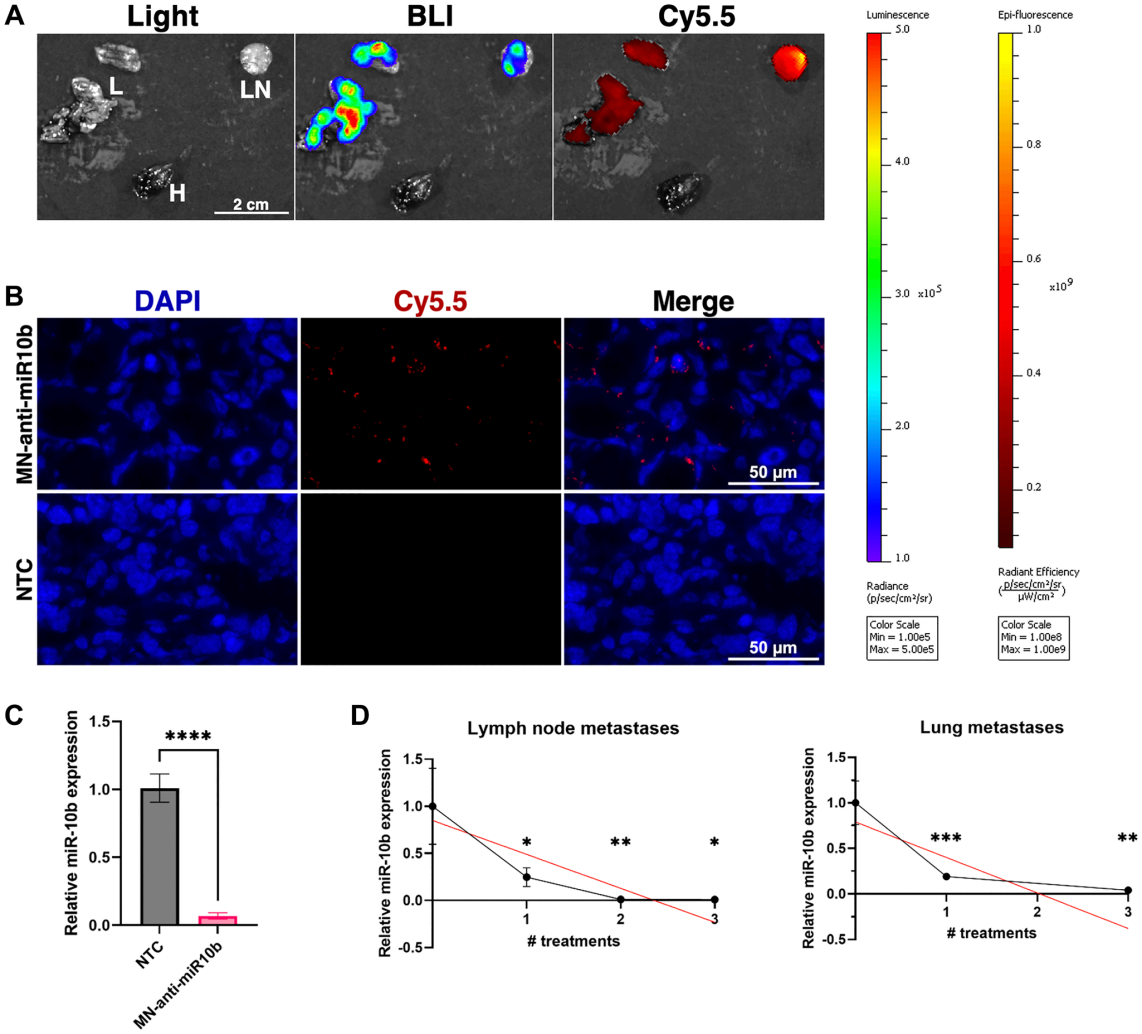
MN-anti-miR10b accumulates in breast cancer metastases within 72 hours and downregulates miR-10b significantly following systemic administration *in vivo*. (**A**) *Ex vivo* imaging of representative metastatic tissues of a mouse treated with MN-anti-miR10b 72 hours prior. Abbreviations: L: lung; LN: lymph node; H: heart. (**B**) Fluorescence microscopy of representative metastatic lymph node tissue treated with MN-anti-miR10b 72 hours prior or non-treated control (NTC). Blue = DAPI. Red = Cy5.5, conjugated to MN-anti-miR10b. (**C**) qPCR of metastatic tissues treated with MN-anti-miR10b 72 hours prior vs. non-treated control (NTC). (**D**) qPCR of metastatic tissues treated with MN-anti-miR10b at weekly intervals, collected 1 week after last treatment. Plots represent mean ± SEM. ^*^ *p* < 0.05, ^**^ *p* < 0.01, ^***^ *p* < 0.001, ^****^ *p* < 0.0001.

We have previously demonstrated that miR-10b expression was significantly inhibited in metastatic lymph nodes 24–48 hours after treatment with MN-anti-miR10b [7, 9]. Here we extended these studies to investigate the time course of the inhibition after repeated treatments in local and distant metastases. To that end, we first confirmed accumulation of the nanodrug in various metastatic sites 72 hours after injection (Figure 1A).

Bioluminescence imaging (BLI) and fluorescence imaging (FLI) of excised lung and lymph node metastases showed excellent co-localization of the nanodrug accumulation with metastatic tissues (Figure 1A) similar to our previous findings [10], which was confirmed by fluorescence microscopy (Figure 1B). Consequentially, RT-qPCR of cryosectioned samples showed that miR-10b was inhibited by over 93% (Figure 1C).

To determine whether miR-10b inhibition remained stable over the course of the repeated treatment, we performed weekly dosing as we did in our previous therapeutic studies [7, 9, 10]. At each treatment point, we tested miR-10b expression in lymph node (LN) and lung metastases. In this study, we found that for each number of treatments and in both lymph node and lung metastases, miR-10b was significantly decreased relative to untreated mice, with downregulation of 99% or greater in lymph node metastases after 2 and 3 treatments and downregulation of over 80% in lung metastasis already after one treatment (Figure 1D). When looking at changes over the course of three treatments, linear regression indicates that miR-10b is gradually decreasing (LNs *p* < 0.021, lung *p* < 0.022). This suggests that the rapid downregulation seen at 72 hours here or in our previous work at 24-48 hours [7, 9] could be transient and that repeated treatments are necessary for stable inhibition of miR-10b.

### MN-anti-miR10b upregulates genes associated with developmental processes

**Figure 2:**
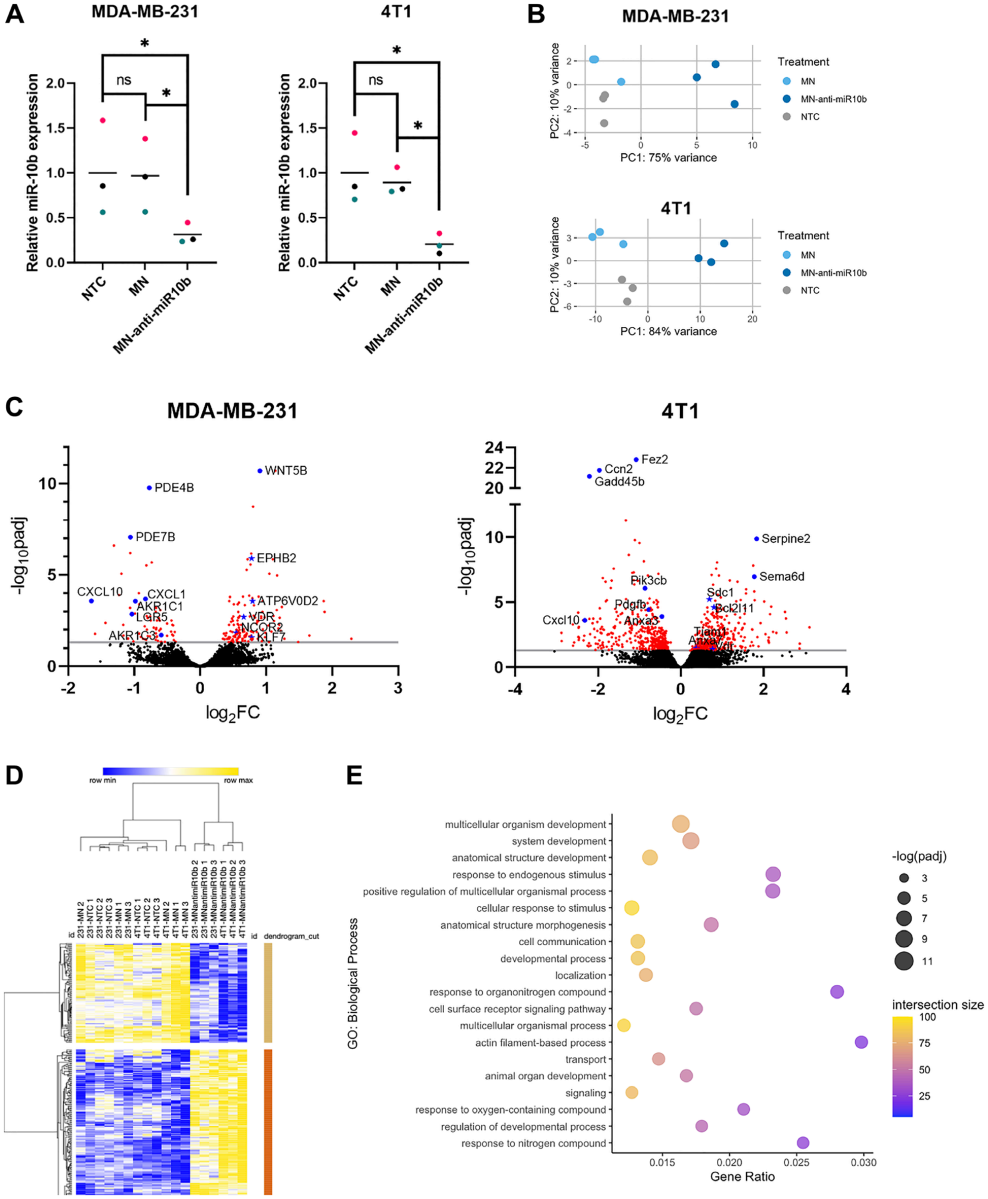
Differential gene expression and functional enrichment analysis of breast cancer cells with miR-10b inhibited by MN-anti-miR10b. (**A**) qPCR of MDA-MB-231 and 4T1 cells treated with MN-anti-miR10b or MN for 48 hours or non-treated control (NTC). Colors represent matched replicates. Line represents mean. ^*^ *p* < 0.05. (**B**) Principal component analysis of transcriptomes of MDA-MB-231 and 4T1 cells treated with MN-anti-miR10b, MN for 48 hours or non-treated control (NTC). (**C**) Volcano plots of MDA-MB-231 and 4T1 cells representing differential gene expression between MN-anti-miR10b and MN treatment for 48 hours. Line indicates *p*_adj_ = 0.05. Red points indicate *p*_adj_ < 0.05. Blue points are notable genes, with titles. Blue stars are predicted targets of miR-10b, with titles. (**D**) Unsupervised hierarchical clustering of normalized, batch-corrected MDA-MB-231 and 4T1 transcriptomes. Rows represent differentially expressed genes between MN-anti-miR10b and MN. (**E**) Top 20 most significant biological processes overrepresented by genes upregulated in MN-anti-miR10b-treated samples vs. MN-treated.

To understand what mechanisms may underlie the therapeutic effects of MN-anti-miR10b, we performed RNA sequencing and differential gene expression analysis on the two triple-negative breast cancer cell lines used in our previous therapeutic studies – human MDA-MB-231 and murine 4T1. Cells were treated with MN-anti-miR10b, vehicle control (MN), or left untreated (non-treated control, NTC) for 48 hours. As both MN-anti-miR10b and MN are routinely synthesized in small batches, successful inhibition of miR-10b by MN-anti-miR10b and not by MN was confirmed prior to sequencing (Figure 2A; *n* = 3 biological replicates). Principal component analysis (PCA) revealed that, in both cell lines, the anti-miR-10b ASO was the largest contributor to variance, as the MN and NTC samples clustered together and apart from MN-anti-miR10b along the first principal component (PC1; Figure 2B). In addition to demonstrating the largest perturbance caused by the nanodrug, importantly, this finding supported MN as a transcriptionally innocuous platform for delivery of therapeutic biomolecules.

With consideration to the differences in human and mouse genomes, we first analyzed the MDA-MB-231 and 4T1 samples independent of each other for differentially expressed genes (DEGs). To identify changes induced by the anti-miR-10b ASO specifically, we compared the transcriptomes of MN-anti-miR10b-treated samples to MN-treated samples. In the MDA-MB-231 samples, we found 144 genes upregulated and 67 genes downregulated by MN-anti-miR10b (Figure 2C). As microRNAs inhibit translation of their target genes [13], upregulation of targets is the most immediate consequence of miR-10b inhibition. Of the upregulated genes, 7 are predicted to be targets on microRNA Target Prediction Database (miRDB) [11] – ATP6V0D2, EPHB2, KLF4, KLF7, NCOR2, TMEM268, and VDR – positioning them as genes whose relationship with miR-10b should be further investigated in the context of miR-10b inhibition-based therapy for metastatic breast cancer. Notable downregulated genes include members of the aldo-keto reductase family 1 (AKR1B1P7, AKR1C1, AKR1C2, AKR1C3), chemokine ligands (CXCL1, CXCL8, CXCL10), and the intestinal stem cell marker LGR5. In the 4T1 samples, more predicted targets were observed among the upregulated genes: Anxa7, Arg2, Bcl2l11, Btbd11, Csgalnact1, Lss, Sdc1, Tiam1, Tnrc6b, Vdr, and Wdr26. Notably, of 10 genes upregulated in both cell lines, VDR/Vdr is the only predicted target.

For a broader approach to understanding the therapeutic mechanisms of miR-10b inhibition, we next sought to identify and compare the biological processes affected by MN-anti-miR10b in the two cell lines. Functional enrichment analysis was performed on upregulated and downregulated genes to determine overrepresented biological processes. Of 72 biological processes overrepresented in genes upregulated by the nanodrug in MDA-MB-231 cells, 60 were also observed in the 4T1 cells (Supplementary Table 1), with many of them relating to developmental processes, including “cell differentiation” and “tissue development.” While this is not surprising given the importance of miRNAs in early development, the persistence of this relationship in the MDA-MB-231 and 4T1 cells suggests that the cells have de-differentiated into a more stem cell-like state and that MN-anti-miR10b may serve to decrease the stem cell-like features commonly associated with cancer cells, such as chemoresistance [14]. In contrast, there was low overlap in the biological processes overrepresented by downregulated genes, all of them being nonspecific (e.g., “biological regulation” and “response to stimulus”; Supplementary Table 1).

For a more global approach to understanding the therapeutic mechanisms of miR-10b inhibition, we analyzed samples from both cell lines together, correcting for cell line as a covariate. Again, we first isolated differences due to the anti-miR-10b ASO by comparing MN-anti-miR10b-treated samples to MN-treated samples, identifying 162 upregulated genes and 98 downregulated genes (Supplementary Figure 1A). When performing unsupervised hierarchical clustering of all samples (including NTC) using the DEGs, MN-treated and NTC samples clustered together and separate from MN-anti-miR10b-treated samples and subsequently clustered by cell line (Figure 2D), supporting the PCAs and indicating that MN has relatively little effect on the cancer cells as a vehicle. As expected, functional enrichment analysis found that developmental processes were significantly overrepresented by the upregulated genes (Figure 2E). Biological processes overrepresented by the downregulated genes include those associated with stress or immune response, possibly indicating that MN-anti-miR10b functions to decrease tumorigenic inflammation (Supplementary Figure 1B).

To show applicability of our findings to other cancers, we sought to determine whether the DEGs and overrepresented biological processes seen with MN-anti-miR10b in breast cancer can be extended to publicly available data. To that end, we compared our dataset to a published microarray dataset in which miR-10b was inhibited in a glioblastoma multiforme (GBM) cell line, U87, by transducing miR-10b binding sites [15]. In the associated study, inhibition of miR-10b resulted in decreased invasion *in vitro* and smaller tumors *in vivo* compared to controls, as seen with our nanodrug [7] and other miR-10b inhibition studies [16].

**Figure 3:**
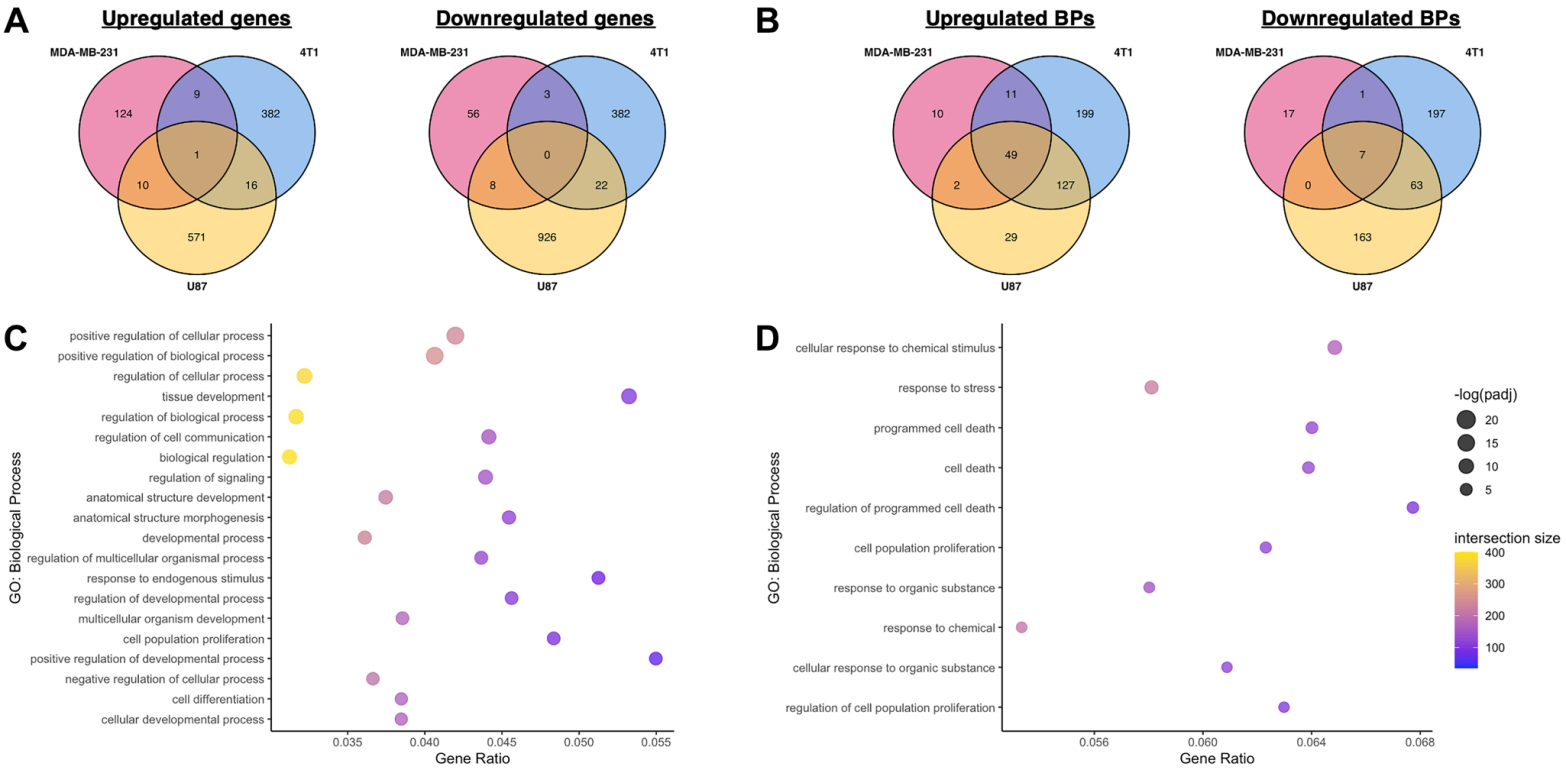
Comparison of transcriptomic effects of miR-10b inhibition in breast cancer cells and glioblastoma. (**A**) Comparison of overlapping genes between MDA-MB-231, 4T1, and U87 datasets, miR-10b inhibited vs. control. (**B**) Comparison of overlapping biological processes overrepresented by upregulated and downregulated genes between MDA-MB-231, 4T1, and U87 datasets, miR-10b inhibited vs. control. (**C**) Top 20 most significant biological processes overrepresented by genes upregulated in U87 dataset, miR-10b inhibited vs. control, also found in MN-anti-miR10b-treated samples. (**D**) All biological processes overrepresented by genes downregulated in U87 dataset, miR-10b inhibited vs. control, also found in MN-anti-miR10b-treated samples.

Comparison of the genes upregulated by miR-10b inhibition identified only one overlapping gene: VDR (Figure 3A). The consistency with which VDR is upregulated in response to inhibition of miR-10b regardless of species, cancer type, or method and its status as a predicted target of miR-10b makes the gene particularly interesting for future studies in miR-10b-based therapies. In contrast, no genes were consistently downregulated in all three cell lines. With consideration to differences between human (MDA-MB-231 and U87) and mouse (4T1) genomes and biology, we further investigated the overlapping DEGs between MDA-MB-231 and U87 cells. In addition to VDR, there were 10 shared upregulated genes (Figure 3A), including the predicted target ATP6V0D2 and two genes within the same family as predicted targets, LRRC8E and WNT5B (LRRC8B and WNT9B are predicted targets), suggesting that these genes may be human-specific effectors of miR-10b inhibition.

Functional enrichment analysis of the upregulated and downregulated genes in the U87 dataset was also performed, and the overrepresented biological processes of each gene set were compared to the ones identified in MDA-MB-231 and 4T1 cells (Figure 3B) and combined analysis (Figure 3C, 3D). Interestingly, despite the low similarity in DEGs, 47 of the 125 biological processes overrepresented by the genes upregulated by MN-anti-miR10b were also overrepresented by the genes upregulated in the U87 dataset (Figure 3C and Supplementary Table 1), again with many of them related to development. From the downregulated genes, despite zero shared genes, 10 overrepresented biological processes are shared (Figure 3D), including processes relating to both cell death and proliferation.

Together, these datasets provide insights into the effects of miR-10b inhibition on cancer cells across delivery methods, tissue types, and species. The relatively large overlap in biological processes relative to the overlap in DEGs suggest that the mechanism of therapeutic efficacy of MN-anti-miR10b may be better explained by its functional effect on cancer cells rather than its effects on any one target or pathway. Specifically, the numerous developmental processes implicated by the upregulated genes continue to support a connection between miR-10b inhibition and induction of differentiation in cancer cells.

### miR-10b is upregulated in cancer cells with increased stemness

**Figure 4:**
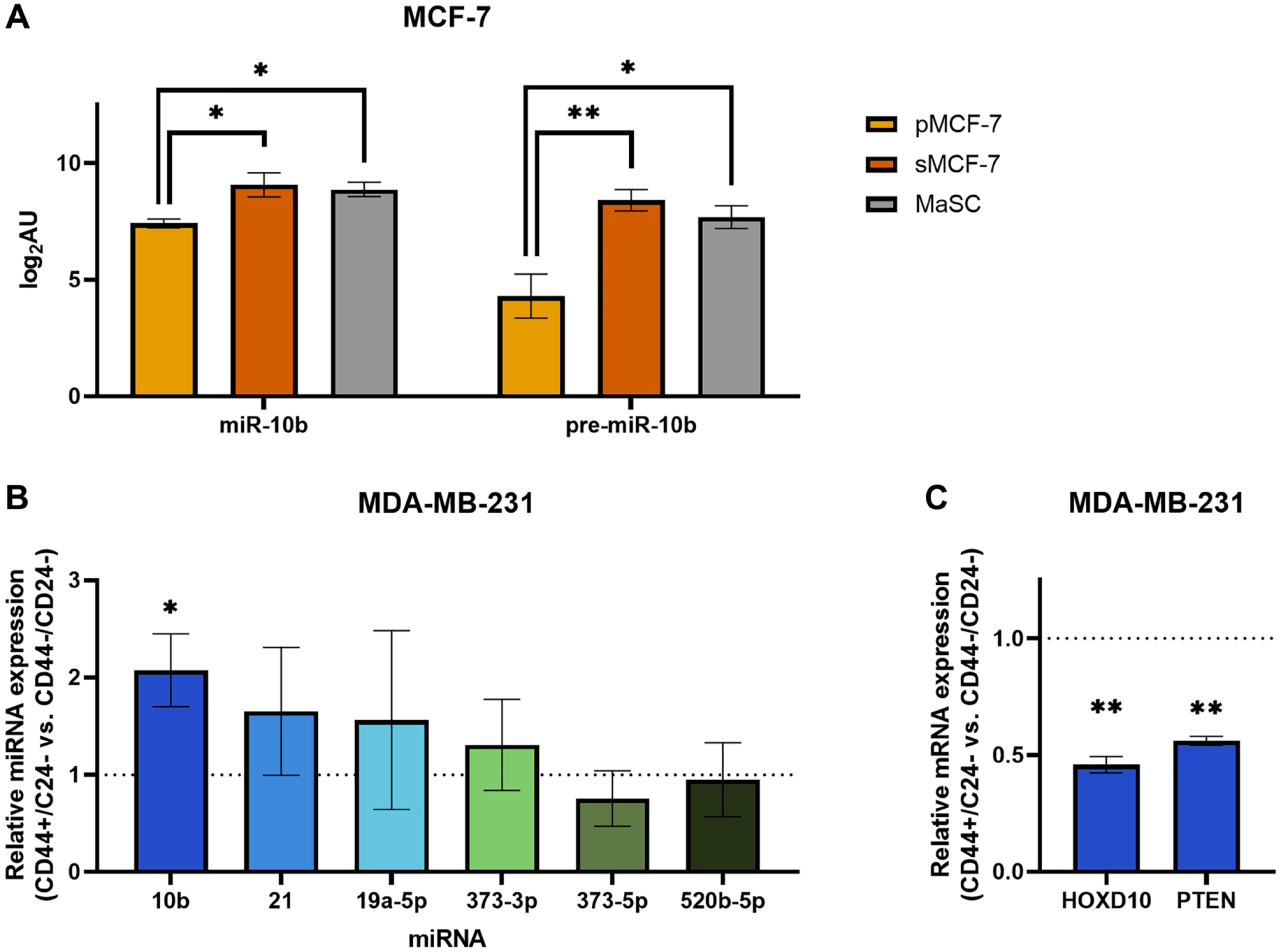
Breast cancer cells sorted for stemness-associated surface markers have upregulated miR-10b expression. (**A**) Log2-transformed arbitrary units of miR-10b and pre-miR10b microarray data in MCF-7 cells. Abbreviations: pMCF-7: parental MCF-7; sMCF-7: sorted (CD44^+^/CD24^–/low^/ESA^+^) MCF-7; MaSC: mammary stem cell. ^*^ *p*_adj_ < 0.05, ^**^ *p*_adj_ < 0.01 (**B**) qPCR of miRNAs representing fold change of CD44^+^/CD24^–^ MDA-MB-231 cells relative to CD44^–^/CD24^–^ (dashed line). (**C**) qPCR of mRNAs representing fold change of CD44^+^/CD24^–^ MDA-MB-231 cells relative to CD44^–^/CD24^–^ (dashed line). B and C: Plots represent mean ± SEM. ^*^ *p* < 0.05, ^**^ *p* < 0.01.

Recent studies have described a link between miR-10b and stem-like properties in cancer cells [17, 18], supporting inhibition of these properties as a possible mechanism for the therapeutic effects of MN-anti-miR10b. To further investigate this relationship, we analyzed publicly available microRNA profiles of MCF-7 breast cancer cells sorted for a surface marker phenotype commonly associated with increased stemness, CD44^+^/CD24^–/low^/ESA^+^ [19–22]. The sorted cells in the dataset were found to have increased tumor initiation capability relative to parental MCF-7 cells and the capacity to differentiate into both epithelial and myoepithelial subpopulations in a tumor [19]. Despite our *a priori* hypothesis that the sorted cells would have greater miR-10b expression, we utilized an unbiased approach to our analysis and applied the Benjamini-Hochberg procedure to the results, finding that sorted CD44^+^/CD24^–/low^/ESA^+^ cells (sMCF-7) have increased miR-10b expression relative to parental MCF-7 cells (pMCF-7) (Figure 4A; *p*_adj_ < 0.022). These results support a study in which MCF-7 cells sorted for only CD44^+^ had increased miR-10b relative to CD44^-^ cells [17]. Additionally, rno-miR-10b is upregulated in the sorted MCF-7 cells (*p*_adj_ < 0.007). In the human genome, the sequence corresponding to rno-miR-10b aligns with the precursor to miR-10b (pre-miR-10b). When comparing these microRNA profiles to the microRNA profile of mammary stem cells (MaSC) [23], although MaSCs have greater miR-10b and pre-miR-10b expression than parental MCF-7 cells (*p*_adj_ < 0.035 and < 0.014, respectively), there are no significant differences between sorted MCF-7 cells and MaSCs, demonstrating the utility of the CD44^+^/CD24^–/low^/ESA^+^ phenotype in selecting for a more stem-like population in breast cancer cells. Indeed, sorted MCF-7 cells and MaSCs cluster together and apart from parental MCF-7 cells when the complete profiles are analyzed by principal component and unsupervised hierarchical clustering (Supplementary Figure 2A).

To translate the relationship between miR-10b and stem-associated properties to our previous studies, we sought to test these findings in MDA-MB-231 cells. Cells were sorted into CD44^+^/CD24^-^ and CD44^–^/CD24^-^ populations, with CD24^+^ cells not analyzed due to consistently low yields. RT-qPCR analysis of the two populations indicate that sorting for CD44 was effective (Supplementary Figure 2B), and subsequent analysis for miR-10b shows that cells with the more stem cell-like CD44^+^/CD24^–^ surface marker phenotype have greater than 2-fold miR-10b expression compared to CD44^–^/CD24^-^ (Figure 4B; *p* < 0.027, *n* = 4 independent sorting events). Notably, of other assorted miRNAs that were tested, none showed significant differences. We then used RT-qPCR to measure relative mRNA expression levels of two targets of miR-10b, HOXD10 [8] and PTEN [24], and observed decreased expression of both genes in the stem cell-like population compared to the non-stem-like population (Figure 4C; *p* < 0.010 and < 0.004, respectively), as would be expected with increased miR-10b expression.

Together, these data support recent claims that miR-10b is associated with cancer cell stemness, importantly demonstrating this relationship in the MDA-MB-231 cell line used in previous studies with the MN-anti-miR10b nanodrug.

### MN-anti-miR10b decreases breast cancer cell stemness

Having identified a correlation between miR-10b expression and a stem-associated surface marker phenotype in both MCF-7 and MDA-MB-231 cells, we next sought to test whether inhibition of miR-10b using MN-anti-miR10b can inhibit properties associated with stem-like cancer cells. *In vitro* methods for studying these properties were reviewed in 2018 by Samanta and Semenza [25].

**Figure 5:**
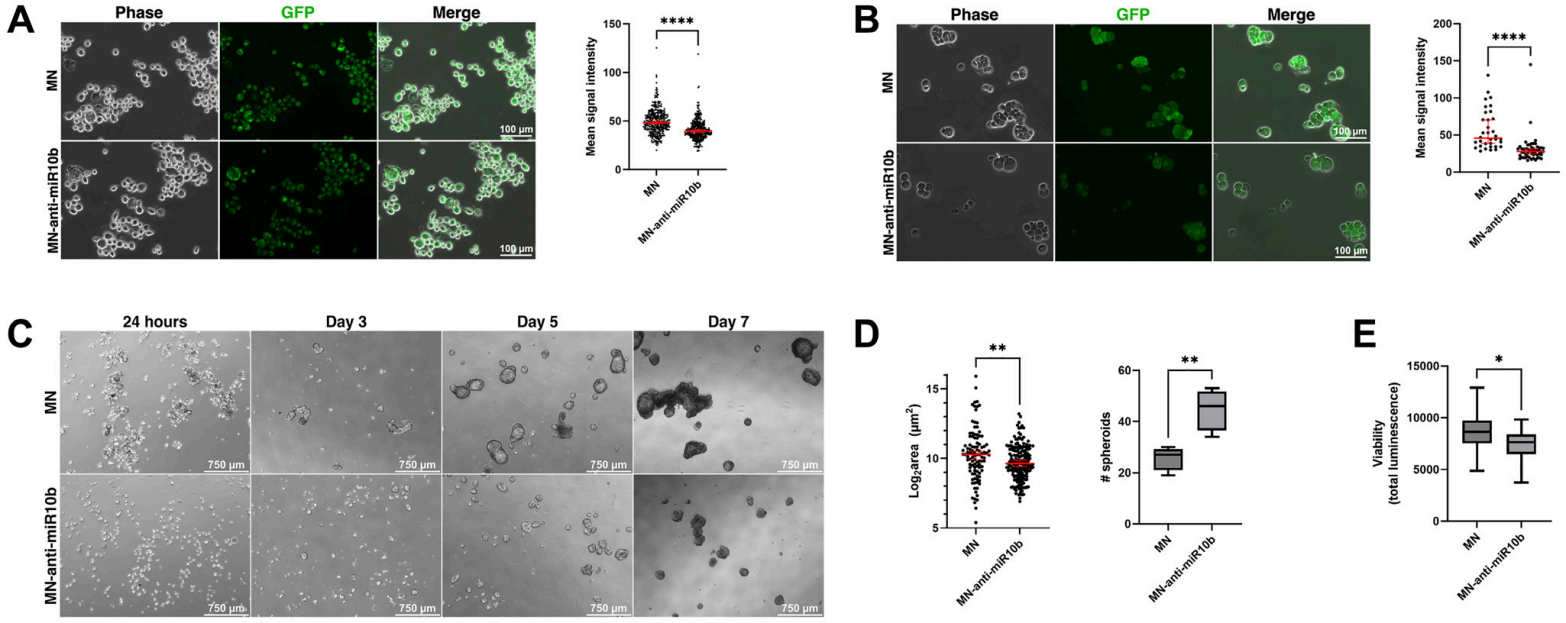
miR-10b inhibition by MN-anti-miR10b decreases Aldefluor accumulation and impairs spheroid formation. (**A**) Fluorescence microscopy of representative MDA-MB-231 cells after incubation with Aldefluor reagent. (**B**) Fluorescence microscopy of representative MCF-7 cells after incubation with Aldefluor reagent. A and B: Mean signal intensity = mean gray value in ImageJ. (**C**) Mammosphere formation over time of MCF-7 cells treated with MN-anti-miR10b or MN 48 hours prior to (adherent conditions) and during culture in mammosphere medium. (**D**) Spheroid size (left) and quantity in a field of view (right) of MCF-7 spheroids at Day 7 in treated medium. Plots represent mean ± SEM (left) and mean ± max/min (right). (**E**) Viability assay of MCF-7 spheroids at Day 7 in treated medium. Plot represents mean ± max/min. A-E: ^*^ *p* < 0.05, ^**^ *p* < 0.01. ^****^ *p* < 0.0001.

The Aldefluor assay is commonly used to identify cancer cells with increased stemness [26]. It reports on the activity of aldehyde dehydrogenase (ALDH), an enzyme known to be overexpressed by stem-like cancer cells and one whose expression selects for subpopulations with increased self-renewal, differentiation, and tumor initiation [27, 28]. The Aldefluor assay of the cells treated with MN-anti-miR10b or MN (vehicle control) for 48 hours revealed that both MDA-MB-231 (Figure 5A) and MCF-7 (Figure 5B) cells treated with MN-anti-miR10b have decreased ALDH activity compared to cells treated with MN (*p* < 0.0001 in both cell lines), suggesting that inhibition of miR-10b reduced stemness. Notably, stemness associated with ALDH activity is reported to be distinct from stemness associated with the CD44^+^/CD24^–^ surface marker phenotype, demonstrating generalizability of the link between miR-10b and different markers of stemness [22].

We further tested our hypothesis by assessing spheroid formation of MCF-7 cells in tumorsphere medium. Tumorsphere formation is indicative of self-renewal capability [29, 30], a characteristic of stem-like cancer cells, and decreased or impaired spheroid formation would support the previous conclusion that inhibition of miR-10b reduces cancer cell stemness. Cells were treated with MN-anti-miR10b or MN for 48 hours under standard, adherent cell culture conditions before being transferred as a single-cell suspension into treated tumorsphere medium in 6-well and 96-well formats for microscopy and a viability assay, respectively. This pre-treatment was done to ensure nanodrug distribution to all cells prior to spheroid formation, as the three-dimensional spheroid structure is known to create a nutrient, oxygen, and drug penetrance gradient [31]. Once transferred to tumorsphere medium, cells were monitored for spheroid formation daily for 7 days. By Day 7, cells treated with MN formed large, clustering spheroids as untreated MCF-7 cells are known to do [30, 32, 33] (Figure 5C). In contrast, cells treated with MN-anti-miR10b form significantly smaller spheroids (less than 38% average surface area as MN, *p* < 0.007), albeit at greater numbers (*p* < 0.007) (Figure 5D). A viability assay performed on Day 7 found that cells treated with MN-anti-miR10b have decreased viability (Figure 5E; less than 14% decrease, *p* < 0.034) relative to cells treated with MN. Notably, staining for dead cells using propidium iodide found no dead cells among cells treated with MN-anti-miR10b (Supplementary Figure 3A), suggesting that the decrease in viability in cells treated with MN-anti-miR10b may be due to reduced proliferation or self-renewal rather than induced apoptotic processes. These experiments were also performed with MDA-MB-231 cells; however, these cells are known to form spheroids poorly [30], aggregating into a loosely-packed structure [34]. As with MCF-7 cells, treatment with MN produced the typical structure of MDA-MB-231 spheroids and treatment with MN-anti-miR10b disrupted this organization (Supplementary Figure 3B), though with no significant effect on cell viability (Supplementary Figure 3C). Thus, these findings were consistent across two cell lines and two characterization methods and indicate that MN-anti-miR10b reduces breast cancer cell stemness.

## DISCUSSION

Inhibition of miR-10b has been shown to be a viable strategy for treatment of metastatic breast cancer [7, 9, 10]. Previously, we have shown that MN-anti-miR10b affects cell migration, invasion, proliferation, and viability with 80–90% downregulation [7, 9]. To understand the effects of the nanodrug and its therapeutic effects over the course of therapy, we first assessed the efficacy with which MN-anti-miR10b downregulates miR-10b *in vivo* and found that the nanodrug decreases expression by 99% after two rounds of weekly treatment, demonstrating comparable if not superior inhibition of miR-10b *in vivo* as is seen *in vitro*. Importantly, we confirmed that this effect was similar in regional (lymph node) and distant (lung) metastases. To investigate secondary effects of miR-10b inhibition by MN-anti-miR10b and to understand the affected pathways, we used RNA sequencing to identify differentially expressed genes and observed an overrepresentation of upregulated genes associated with developmental processes, suggesting an effect on the stem cell-like properties of cancer cells. We then confirmed that miR-10b is associated with cancer cell stemness and that phenotypes associated with stemness could be mitigated by MN-anti-miR10b. Together, these data indicate that MN-anti-miR10b has a differentiation effect on cancer cells and implicate dedifferentiated, stem cell-like cancer cells as most vulnerable to its action. This could also explain why in our earlier studies treatment of the primary MDA-MB-231 tumors with the nanodrug completely abrogated metastasis formation [7], as these metastasis-forming stem cell-like cancer cells lost their ability to invade and migrate and most likely died within the primary tumor.

The upregulation of genes associated with developmental processes by MN-anti-miR10b is not unexpected. While details of the role of miR-10b beyond cancer are sparse, miRNAs are collectively associated with regulation of growth and development [35]. Furthermore, the effects of miRNAs are influenced in part by their location in the genome and miR-10b is coded among the HOXD cluster of genes [36]. Indeed, HOXD10 was one of the first genes found to be regulated by miR-10b [8] and is a computationally predicted target [11]. The finding is notable, though, as it suggests that the cancer cells overexpressing miR-10b are in a less-developed, more stem cell-like state. This is supported by previous findings that more mesenchymal cancer cell lines have higher susceptibility to MN-anti-miR10b than more epithelial cell lines [9], as mesenchymal cancer cells share many of the same properties as stem-like cancer cells and the epithelial-mesenchymal spectrum is commonly associated with the spectrum of stemness [37, 38]. Additionally, higher susceptibility to MN-anti-miR10b has been seen in cancer cell lines with increased expression of genes associated with the proto-oncogene transcription factor c-Jun [12]. As c-Jun has been implicated in conferring stemness in cancer cells [39, 40], this further supports the notion that cancer cells with increased stemness are most sensitive to the nanodrug. Indeed, there are no indications of toxicity from MN-anti-miR10b in developed tissues [7, 9].

Evidence for stem-like states in cancer cells date back to 1994, when a subpopulation of acute myeloid leukemia (AML) cells was found capable of inducing AML in mice when other subpopulations could not [41]. A stem cell-like state in cancer cells is thought to be achieved through either transformation of an adult stem cell or through dedifferentiation of a malignant cell [42]. In this state, the cancer cells have numerous properties that allow them to evade complete eradication. For example, their capacity to self-renew allows for increased tumor initiation capability, whether in the form of primary tumors or metastases, and their ability to differentiate confers tumor heterogeneity [43]. Furthermore, the cells reside in a metabolically quiescent state [14], allowing them to resist therapeutics aimed at rapidly dividing cells. Differentiation therapy to decrease these properties is a focus of many modern research efforts, buoyed by the successful use of all-trans retinoic acid in treating acute promyelocytic leukemia [44]. In solid tumors, differentiation therapy has also resulted in increased cure rates in neuroblastoma patients [45]. While similar successes have not yet been seen in breast cancer, research is ongoing. Several studies have produced promising results in preclinical experiments [46] (including miRNA-based approaches [47]) and early-stage clinical trials [48], and recent efforts aimed at computationally modeling stemness in breast cancer may uncover novel insights into how to best implement differentiation therapy [49].

Surface markers have been used to identify cells with increased stemness since the aforementioned AML study, in which AML-inducing cells were identified by their CD34^+^/CD38^−^ phenotype [41]. While markers vary across cell lines [50, 51], they are generally validated by testing for similar phenotypic properties, such as increased tumor initiation capacity [50]. The CD44^+^/CD24^-^ phenotype was reported to be a marker of increased stemness in breast cancer in 2003 by Al-Hajj et al. [20]. Many groups have since validated this finding in their research and it continues to be commonly used for the isolation of breast cancer cells with increased stemness [21, 22] and studies into prognostic indicators (reviewed in [52]). In this study, we showed that in two breast cancer cell lines – MDA-MB-231 and MCF-7 – subpopulations with the CD44^+^/CD24^-^ surface marker phenotype have increased miR-10b expression relative to their parental cell line or other subpopulations. The stem-like properties of the CD44^+^/CD24^-^ in these cell lines have been described previously [17, 21, 22, 53]. These findings support previous studies that report that miR-10b drives a stem cell-like phenotype in both cancer cells [17, 18, 54] and progenitor cells [55].

The two most common methods for characterizing stemness in cancer cells *in vitro* are the Aldefluor assay and mammosphere formation [25]. The Aldefluor assay has been used to identify cells with increased stemness in both healthy and cancerous contexts, hematopoietic and solid, and increased ALDH activity in cancer cells is associated with properties such as drug resistance, tumorigenicity, and invasiveness [27, 28]. We observed decreased ALDH activity in the cells after treatment with MN-anti-miR10b for 48 hours. Notably, our RNA sequencing studies did not show any significant changes in the gene expression of any ALDH family genes; however, ALDH family member L2 (ALDH1L2) was significantly downregulated in the U87 dataset (probe 231202_at; *p*adj < 0.029). Though these results are incongruent, the sequencing results are only indicative of transcript expression and are not necessarily indicative of the activity of ALDH protein. Indeed, ALDH enzymes are reported to have low turnover rates [56], and thus, changes in transcript expression should not be expected in a 48-hour treatment period. In contrast to the Aldefluor assay, mammosphere assays characterize stemness using the ability of cells to self-renew and form three-dimensional spheroids in anchorage-independent conditions [29]. Cells grown in these conditions display increased drug resistance, proliferation, and migration properties [30]. Notably, patient-derived metastatic cells are more effective at forming mammospheres than cells isolated from primary tumors [57]. In our mammosphere assays, we found that treatment with MN-anti-miR10b prevented MCF-7 cells from forming large spheroids, supporting similar studies that inhibited miR-10b by other means [17, 18]. Of note, the reverse relationship wherein miR-10b increases spheroid size has also been observed [17]. Phenotypic effects were also seen in MDA-MB-231 cells, though their analysis is limited by their poor mammosphere formation. This is believed to be only structural and not functional, as MDA-MB-231 cells grown in mammosphere medium display the same enhanced stem cell-like properties as other cancer cell lines when grown in mammosphere medium [30]. Collectively, our results demonstrate that inhibition of miR-10b using MN-anti-miR10b decreases the stemness of breast cancer cells, supporting dedifferentiation as a mechanism through which the nanodrug may function as a therapy. In addition, these findings may be significant for synergizing anti-miR10b nanodrug with a standard of care in first-in-human clinical trials where testing it as a monotherapy is not likely. Finally, given the proven role played by miRNA-10b in other cancers beyond breast cancer, including lung, colorectal, gastric, bladder, pancreatic, ovarian, hepatocellular and brain cancer [58–60], these findings could have broad implications for the treatment of metastatic carcinoma in general.

## MATERIALS AND METHODS

### MN-anti-miR10b (nanodrug) synthesis and characterization

The MN-anti-miR-10b was prepared following previously established protocol [9]. Briefly, first the magnetic nanoparticle (MN) core was prepared by co-precipitation method and then it was aminated. The aminated MN have hydrodynamic diameter of 24.3 nm with 90 amines per MN particle. The aminated MN was then labeled with Cy5.5 nearinfrared optical dye by reacting with Cy5.5-NHS ester (Lumiprobe). For conjugation of MN-Cy5.5 with anti-miR-10b locked nucleic acid (LNA, Integrated DNA Technologies), MN-Cy5.5 was activated with heterobifunctional linker N-succinimidyl 3-[2-pyridyldithio]-propionate (SPDP; Thermo Fisher Scientific) and 5′-ThioMC6 end of the LNA was activated by treating with 3% TCEP. Finally, SPDP conjugated MN-Cy5.5 was incubated with activated LNA to yield the MN-anti-miR-10b. The conjugation resulted in ~9 LNA oligo per MN particle.

### Cell culture

MDA-MB-231 cells expressing luciferase (MDA-MB-231-luc-D3H2LN; Perkin Elmer) and MCF-7 cells (ATCC) were grown in DMEM supplemented with 10% FBS and antibiotics (100 units/mL penicillin, 100 mg/mL streptomycin). 4T1 cells expressing luciferase (4T1 Red F-luc; Perkin Elmer) were grown in DMEM supplemented with 5% FBS and antibiotics.

### Animal model, treatment, and *in vivo* and *ex vivo* imaging

All procedures involving animal subjects have been approved by the Michigan State University Institutional Animal Care and Use Committee (IACUC) and conformed to all regulatory standards. Eight-week-old female nude mice (nu/nu; Jackson Laboratory, *n* = 24) were orthotopically implanted with 2 × 10^6^ MDA-MB-231 human triple-negative breast cancer cells (50% PBS, 50% Matrigel) under the third mammary fat pad and monitored for metastasis formation using bioluminescence imaging (BLI), as described previously [9]. With a focus on metastatic disease, primary tumors were resected when they began to compromise mouse mobility or became at risk of infection due to severe ulceration, as advised by veterinary staff.

Treatment with MN-anti-miR10b (10 mg Fe/kg bodyweight) via tail vein injection was initiated when metastasis signal reached 1 × 10^5^ radiance (2 cm × 2 cm ROI). Mice were treated a single time with collection after 72 hours, or once per week for up to three weeks and collection one week after each last treatment (i.e., collected one week after one treatment, one week after two treatments and one week after three treatments). Metastatic tissue collection was guided by BLI and nanodrug delivery to the tissue was confirmed using fluorescence imaging in Cy5.5 channel. Tissues were then cryopreserved in OCT for processing.

### Fluorescence microscopy of metastatic tissue sections

Tissues were cryosectioned at 10 μm, fixed in 4% paraformaldehyde for 15 minutes, and mounted using DAPI Fluoromount-G (SouthernBiotech). Slides were imaged using channels for DAPI and Cy5.5 using a Nikon Eclipse 50i fluorescence microscope charge coupled device camera with near-IR sensitivity (SPOT 7.4 Slider RTKE), and SPOT 4.0 Advance version software (Diagnostic Instruments).

### RNA extraction and RT-qPCR

RNA of tissues was extracted from ten 10 μm sections by phenol-chloroform extraction (Qiagen) and further purified using the Zymo Quick-RNA 96 Kit. For *in vitro* samples, RNA was extracted and purified using only the Zymo Quick-RNA 96 kit. Reverse transcription and qPCR were performed using the mir-X miRNA First Strand Synthesis and RT-qPCR TB Green Kits (Takara Bio) for the analysis of miR-10b (TAC CCT GTA GAA CCG AAT TTG TG), U6 (reference gene for miRNAs, included in kit), PTEN (forward TCCTGGATGACCTTTGACATAC, reverse CCAACTTTGGTTTAATGCACAAC), HOXD10 (forward CGATTTATGCCTTGTAGCCTTTC, reverse GCATTATACATGCGACCAGAAC), and 18S (reference gene for mRNAs; forward CCAGTAAGTGCGGGTCATAAG, reverse GGCCTCACTAAACCATCCAA).

### Cell treatment

Human triple-negative breast cancer cells MDA-MB-231, murine triple-negative breast cancer cells 4T1, and ER and PR-positive human breast cancer cells MCF-7 cells were plated in 6-well plates at an initial plating density of 1 × 10^5^ cells/well and treated 24 hours later using MN-anti-miR10b or MN at 50 μg Fe/mL (approximately 2.4nmol total oligomer), as used in previous *in vitro* studies [7]. Cells were analyzed after 48 hours treatment.

### RNA sequencing and raw data processing

RNA sequencing was performed by the Michigan State University Genomics Core. Sequencing libraries were prepared using the Illumina stranded mRNA library prep kit (Illumina) with IDT for Illumina RNA Unique Dual Index adapters following the manufacturer’s recommendations, except that half-volume reactions were performed. Libraries were assessed for quantity and quality using a combination of Qubit dsDNA HS (Thermo Fisher Scientific) and Agilent 4200 TapeStation HS DNA1000 assays (Agilent). Libraries were pooled in equimolar amounts, and the pool was quantified using an Invitrogen Collibri quantification quantitative PCR kit (Invitrogen). The pooled library was loaded onto two lanes of a NovaSeq SP flow cell, and sequencing was performed in a 1 × 100-bp single-read format using a NovaSeq 6,000 v1.5 100-cycle reagent kit (Illumina). Base calling was performed with Illumina real-time analysis (version 3.4.4), and the output of real-time analysis was demultiplexed and converted to the FastQ format with Illumina Bcl2fastq (version 2.20.0).

RNA sequencing data analysis was supported through computational resources provided by the Institute for Cyber-Enabled Research at Michigan State University. FastQC (version 0.11.7) was used for pre-processing read quality assessment. Read mapping was performed against the GRCm39/mm39 mouse reference genome or the GRCh38 human reference genome, as appropriate, using Bowtie2 (version 2.4.1) with default settings. Read counts were quantified using the FeatureCounts function from the Subread package (version 2.0.0).

These data are publicly available in the Gene Expression Omnibus (GEO), Accession: GSE270229.

### Analysis of RNA sequencing counts files and figure generation

Differential gene expression analysis was performed in R using the DESeq2 package (version 1.42.1) [61]. Principal component analysis and visualization were performed in R using the ggplot2 package (version 3.5.1) [62]. Volcano plots of the differentially expressed genes (DEGs) were produced using GraphPad Prism (version 9.5.0). Unsupervised hierarchical clustering was performed in the web-based Morpheus software interface by the Broad Institute (https://software.broadinstitute.org/morpheus/), using one minus Pearson correlation and average linkage. Functional enrichment analysis for overrepresented biological processes was performed using the web-based g:Profiler interface (https://biit.cs.ut.ee/gprofiler/gost) [63]. Dot plots of overrepresented biological processes were produced using ggplot2. Venn diagrams of DEGs and overrepresented biological processes were produced using the VennDiagram package (version 1.7.3) [64].

### Public microarray datasets and analysis

Gene expression profiles of U87 cells transduced with miR-10b binding sites are publicly available in the GEO under the accession number GSE35170 [15], and miRNA profiles of sorted MCF-7 and mammary stem cell populations are publicly available under the accession number GSE68271 [19, 23]. Analysis of the datasets was performed in R using the limma package (version 3.58.1) [65]. For the MCF-7 dataset, units were log2-transformed prior to analysis.

### Surface marker-based live cell sorting

MDA-MB-231 cells were sorted using the MojoSort system, PE anti-human CD44 and APC anti-human CD24 antibodies, and MojoSort Human anti-PE and anti-APC nanobeads (BioLegend). Cells were first sorted for CD24, yielding CD24^+^ and CD24^-^ populations. This yielded relatively low numbers of CD24^+^ cells; thus, they were not further processed or analyzed. The CD24^-^ population was subsequently sorted for CD44, yielding the CD44^+^/CD24^-^ and CD44^-^/CD24^-^ populations used in RT-qPCR analysis.

### Aldefluor assay

Aldefluor assay was performed using the ALDEFLUOR kit (STEMCELL Technologies), with modifications to allow for microscopic analysis, as has been demonstrated previously [66]. Cells were initially prepared according to manufacturer instructions, with cell concentrations of 1.5 × 10^6^ cells/mL for MDA-MB-231 cells and 5 × 10^5^ cells/mL for MCF-7 cells, using activated reagent at a concentration of 5 μL reagent/1 mL cells, and incubating for 30 minutes. Cells were then pelleted at 300 × G for 5 minutes. The supernatant was aspirated, and the pellet was resuspended in 50 μL of Aldefluor buffer. Ten microliters of cell suspension was transferred onto a microscope slide, coverslipped, and imaged using Phase contrast and the GFP light cube on the EVOS M5000 microscope. Fluorescence intensity of cells was analyzed using Fiji/ImageJ. Briefly, the Phase image was converted into regions of interest (ROI) approximating individual cells using the Hough Circle Transform plugin (UCB Vision Sciences). These ROIs were then applied to the corresponding GFP images and measured for mean gray value.

### Spheroid formation, viability, and propidium iodide staining

MDA-MB-231 and MCF-7 cells were treated with MN-anti-miR10b or MN for 48 hours under standard, adherent conditions before being transferred to treated 3D Tumorsphere Medium XF (PromoCell) and ultra-low attachment plates (Corning). Spheroid growth over time was imaged using the EVOS M5000 microscope. Spheroid surface areas and number of spheroids were measured using ImageJ. Viability assays were performed using CellTiter-Glo (Promega), as it is lytic and best suited for analysis of spheroids [67], measuring total luminescence. Propidium iodide (PI; Thermo Fisher Scientific) staining was performed by adding PI at a final concentration of 5 ng/mL directly to the spheroids in tumorsphere medium 24 hours prior to imaging. Images were taken using the Leica Thunder Imager.

### Statistical analysis

RNA sequencing and microarray data differential gene expression analyses were performed using DESeq2 and limma, as described in the corresponding sections. A simple linear regression was used to analyze time-course data. For all other applications, analysis between experimental and control groups were analyzed using a two-tailed *t*-test. *p* < 0.05 was interpreted as statistically significant.

## SUPPLEMENTARY MATERIALS





## Abbreviations

miR-10b: microRNA-10b
ASOs: antisense oligomers
MN: magnetic nanoparticles
MRI: magnetic resonance imaging
FLI: fluorescence imaging
BLI: bioluminesce imaging
LN: lymph node
NTC: non-treated control
DEGs: differentially expressed genes
GBM: glioblastoma multiforme
sMCF-7: sorted CD44^+^/CD24^–/low^/ESA^+^ cells
pMCF-7: parental MCF-7 cells
MaSC: mammary stem cells
ALDH: aldehyde dehydrogenase
AML: acute myeloid leukemia
