# Supplementary material for: Inhibition of miR-10b treats metastatic breast cancer by targeting stem cell-like properties
Source: Oncotarget. 2024 Aug 26;15:591–606. doi: 10.18632/oncotarget.28641 (PMC11348941; doi:10.18632/oncotarget.28641)
Supplement: Supplementary file 1 [file oncotarget-15-28641-s001.pdf]

Inhibition of miR-10b treats metastatic breast cancer by targeting stem cell-like properties

SUPPLEMENTARY MATERIALS

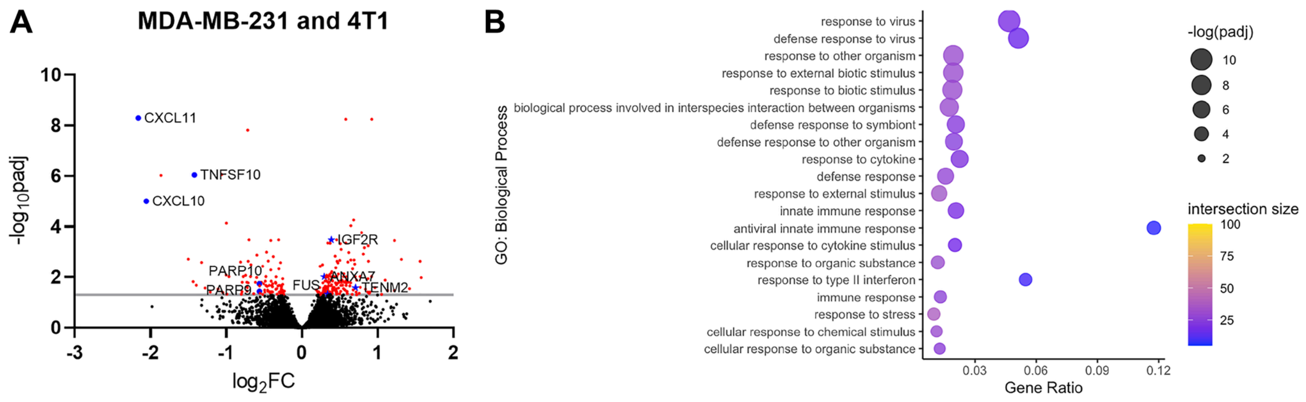

Supplementary Figure 1: (**A**) Volcano plot representing differential gene expression between MN-anti-miR10b and MN treatment for 48 hours, combined MDA-MB-231 and 4T1 datasets. Line indicates $p_{adj} = 0.05$. Red points indicate $p_{adj} < 0.05$. Blue points are notable genes, with titles. Blue stars are predicted targets of miR-10b, with titles. (**B**) Top 20 most significant biological processes overrepresented by genes downregulated in MN-anti-miR10b-treated samples vs. MN-treated.

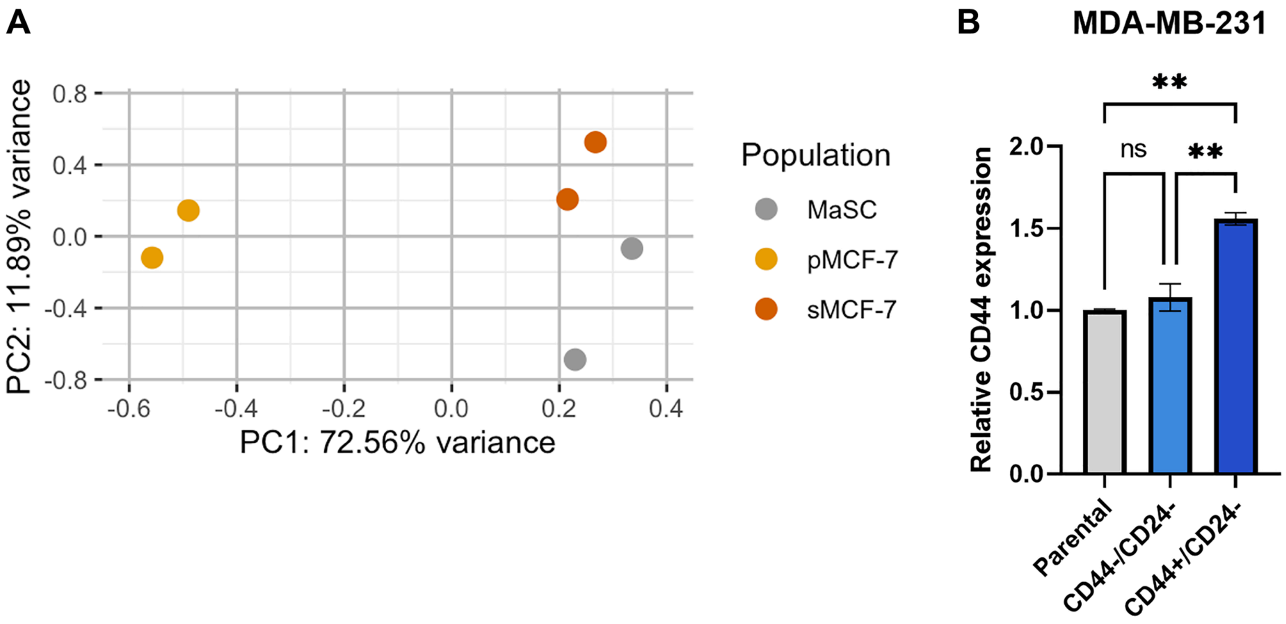

Supplementary Figure 2: (**A**) Principal component analysis of miRnomes of parental (*p*) and sorted (s) MCF-7 cells and mammary stem cells (MaSC). (**B**) qPCR of CD44 for $CD44^{+}/CD24^{-}$, $CD44^{-}/CD24^{-}$, and parental MDA-MB-231 cells. Plot represents mean ± SEM. $^{*}p < 0.05$, $^{**}p < 0.01$.

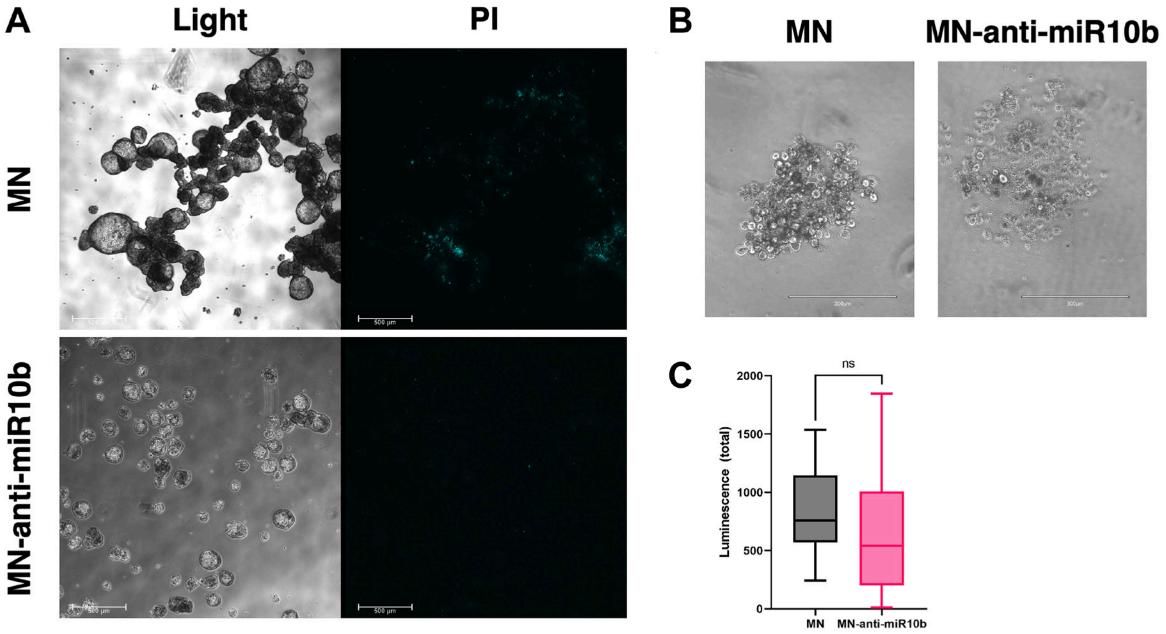

Supplementary Figure 3: (**A**) Light and fluorescence microscopy for PI (cyan) of MCF-7 cells treated with MN-anti-miR10b or MN 48 hours prior to (adherent conditions) and during culture in mammosphere medium. (**B**) Mammosphere formation at Day 11 of MDA-MB-231 cells treated with MN-anti-miR10b or MN 48 hours prior to (adherent conditions) and during culture in mammosphere medium. (**C**) Viability assay of MDA-MB-231 spheroids at Day 11 in treated medium. Plot represents mean ± max/min. Abbreviation: ns: not significant.

Supplementary Table 1: Biological processes overrepresented by genes downregulated or upregulated by miR-10b inhibition. See Supplementary Table 1.
